# Comment on “Radiation protection: safety measures and knowledge among interventional radiologists – a UK-based analysis of current practices and recommendations for improvement”

**DOI:** 10.1186/s42155-025-00632-0

**Published:** 2025-12-13

**Authors:** Bahman Rasuli

**Affiliations:** Department of Radiology, Jame Jam Imaging Center, Tehran University of Medical Sciences, Tehran, Iran

Dear Editor,

We read with great interest the recent article by Gasiea et al. entitled “Radiation protection: safety measures and knowledge among interventional radiologists — a UK-based analysis of current practices and recommendations for improvement,” published in *CVIR Endovascular*. The topic of radiation safety is of undeniable importance, and we commend the authors for highlighting this essential issue within the interventional radiology community.

Nevertheless, several methodological aspects warrant further discussion to strengthen the interpretation of their findings.

First, the study reported that 112 of 1100 members of the British Society of Interventional Radiology completed the questionnaire, representing a response rate of approximately 10%. While response rates in online health research surveys can reach 40–45% when optimized recruitment strategies are employed, specialist society surveys particularly within procedural disciplines such as interventional radiology, cardiology, and endovascular surgery typically yield lower participation rates (10–25%) depending on workload and survey design [1, 2]. Even so, limited participation inevitably introduces potential response bias and constrains the representativeness of conclusions.

Second, while the article emphasizes concerns regarding radiation exposure in women and pregnancy, the overall proportion of male-to-female respondents was not provided. Although 23 respondents reported pregnancy while working with radiation, which offers meaningful data on occupational adjustments during pregnancy, providing the full demographic distribution would enhance transparency and enable more robust subgroup comparisons in future surveys.

Third, subgroup analyses comparing interventional radiologists, radiographers, and nurses were based on small numbers (e.g., 9 nurses, 16 radiographers). Although the authors acknowledged this limitation, the finding still underscores the need for larger, nationally coordinated datasets to capture training needs and practice variation more reliably.

Furthermore, recent evidence supports the inclusion of biological endpoints in future radiation safety studies. Investigations have demonstrated increased genomic instability among interventional radiologists chronically exposed to low-dose ionizing radiation [3], as well as persistent ocular risks, with surveys reporting a 16–17% prevalence of radiation-induced cataracts and inadequate use of protective eyewear [4, 5]. Incorporating such objective measures in future research would provide a more comprehensive assessment of occupational exposure effects.

Finally, while the study’s conclusions are based on a limited respondent cohort, they nevertheless highlight genuine awareness gaps and variation in practice, reinforcing the ongoing need for education and institutional support in radiation protection.

We thank the authors for addressing this vital topic and hope these comments contribute to constructive dialogue aimed at advancing occupational safety standards in interventional radiology.

## Data Availability

Not applicable.
